# Fruit and seed attributes of plants derived from crossing between triploid Passionflower cytotypes

**DOI:** 10.1007/s10265-026-01695-3

**Published:** 2026-03-09

**Authors:** Claudinei da Silva Souza, Marcelo Dias Machado, Alana Jeniffer Alves dos Santos, Gabrielle Balbo Crepaldi, Elyabe Monteiro de Matos, Lyderson Facio Viccini, Ilio Fealho de Carvalho, Wagner Campos Otoni, Aryane Campos Reis, Diego Ismael Rocha, Maurecilne Lemes da Silva

**Affiliations:** 1 Programa de Pós-Graduação em Genética e Melhoramento de Plantas, Universidade do Estado de Mato Grosso, Tangará da Serra, MT Brazil; 2 Departamento de Biologia, Instituto de Ciências Biológicas, Universidade Federal de Juiz de Fora, Juiz de Fora, MG Brazil; 3 Faculdade de Ciências Agrárias, Biológicas e da Saúde, Departamento de Biologia, Universidade do Estado de Mato Grosso, Tangará da Serra, MT Brazil; 4 Departamento de Biologia Vegetal, Universidade Federal de Viçosa, Viçosa, MG Brazil; 5 Departamento de Agronomia, Universidade Federal de Viçosa, Viçosa, MG Brazil; 6 Programa de Pós-Graduação em Genética e Melhoramento de Plantas, Departamento de Ciências Biológicas, Universidade do Estado de Mato Grosso, Av. Inácio Bittencourt Cardoso, 6967, Jardim Aeroporto, Cx 287, Tangará da Serra, Mato Grosso 783009-70 Brazil

**Keywords:** Aneuploid, Crossbreeding, Germination, *Passiflora*, Triploid

## Abstract

Triploid plants are usually sterile or produce non-viable seeds, but may produce fruits. This study examined the morphological and reproductive characteristics of triploid *Passiflora cincinnata* Masters, with a focus on fruit and seed production. Triploid plants were obtained through in vitro cultivation of the endosperm via somatic embryogenesis, whereas diploid plants were raised through seed germination. Diploid and triploid plants were confirmed by flow cytometry and cytogenetic analyses. Cross-pollination and self-pollination yielded, on average, 18 fruits per plant in the cytotype diploid, while triploids produced only 6 fruits. Diploid plants have more uniform fruits, while triploid plants showed variations in size. Seeds with malformations or absence of zygotic embryos and endosperm were observed in triploid cytotypes, while remaining below 1.5% in diploid cytotypes. It was observed that the highest germination rate of 80% occurred in seeds of the diploid cytotype, compared to 53.3% in triploids. The seedlings from the cross between diploid plants contained 3.29 pg of nuclear DNA and a chromosome number of 2n = 2x = 18, while the seedlings from the cross between triploid plants had progeny with 3.37 pg of nuclear DNA and variable chromosomes numbers, of 2n = 19 (2n + 1), 2n = 15 (2n − 3), and 2n = 16 (2n − 2) chromosomes confirming aneuploidy in the progeny. Stomatal analysis showed that diploid plants displayed the highest stomatal density, whereas triploid and aneuploid plants had the greatest length and width of stomata. The density and width of pavement epidermal cells varied among cytotypes. Meiotic disturbances in triploid plants cause phenotypic alterations in the progeny, resulting in delayed vegetative development, low vigor, non-uniform germination, and death during the juvenile phase. The genetic material of aneuploids is a resource for gene mapping, enabling the modification or production of new cultivars with economically relevant phenotypes.

## Introduction

In plants, different ploidy levels can be highly beneficial for stabilizing specific characteristics, such as organ size, fruit quality, yield, and environmental adaptation. While polyploid plants present interesting genotypes for crop breeding programs, the use of synthetic polyploids in agriculture remains limited (Bomblies 2020; Herben et al. 2017; Liqin et al. 2019; Ruiz et al. 2020; Rutland et al. 2021; Zhang et al. 2019). New phenotypes may emerge through interactions between transcription factors of duplicated genomes, giving rise to either a new function or retention of the ancestral one (Drapal et al. 2020; Li et al. 2019; Rutland et al. 2021; Spoelhof et al. 2017).

Recently, triploid passion fruit plants have been regenerated via in vitro endosperm cultivation (Antoniazzi et al. 2018; Ramakrishnan et al. 2025; Rocha et al. 2020). Endosperm tissue is triploid in most higher plants, which facilitates the production of polyploid plants (Machado et al. 2005; Otoni et al. 2024). This technique has been established for *Passiflora edulis* (2n = 3x = 27) (Antoniazzi et al. 2018; Tang et al. 2024), the main commercial species of the genus, as well as for *Passiflora cincinnata* Mast. (Machado et al. 2022; Silva et al. 2020) and *Passiflora foetida* L. (Mikovski et al. 2021). In all instances, plants derived from the endosperm exhibited ploidy levels consistent with those of the endosperm, confirming their triploid nature (Mikovski et al. 2019; Narukulla et al. 2024; Soares-Scott et al. 2005). Analysis of morphoagronomic traits in *P*. *cincinnata* (Machado et al. 2022) and *P*. *foetida* (Mikovski et al. 2021) revealed greater vegetative and reproductive organ sizes in triploid plants compared to their diploid counterparts. Although triploid plants are generally sterile, some studies have shown fruit and seed formation in these genotypes (Aleza et al. 2010; Ramakrishnan et al. 2025; Suzuki and Yamagishi 2016; Wang et al. 2023; Zhang and Park 2010).

Reproduction in polyploid plants relies on homologous chromosomes for proper pairing during meiosis. If pairing does not occur, multivalent chromosomes form during prophase I, leading to the production of aneuploid gametes (Ramsey and Schemske 2002). Chromosomal loss or gain can affect morphological, anatomical, cytological, and physiological characteristics (Birchler 2013; Sheltzer et al. 2012; Singh 2020). Aneuploidy is characterized by a deviation from the normal number of chromosomes, either through loss or gain, resulting in an imbalance in gene dosage and severe phenotypic changes (Makarevitch and Harris 2010; Zeng et al. 2020). Aneuploid plants exhibit delayed vegetative development, partial sterility, and premature death (Kaya et al. 2020; Makarevitch and Harris 2010). The use of aneuploids in plant breeding is a valuable resource for genetic mapping, dosage of repetitive genes, and identification of genes and chromosomal regions responsible for quantitative agronomic traits (Birchler 2013; Singh 2020; Wang et al. 2016).

In the *Passiflora* genus, the basic chromosome number is x = 6; however, secondary chromosomal numbers of x = 9, 10, and 12 have been observed (Melo et al. 2001), along with naturally occurring polyploid and aneuploid chromosomal numbers, such as 2n = 14, 20, 22, 24, 36, 72, and 84. For example, aneuploidy in *P*. *foetida* leads to 2n = 18, 20, 22, and 28 (Soares-Scott et al. 2005).

In this study, we aimed to investigate the morphology of triploid *P*. *cincinnata* plants, as well as their fruit and seed production. *Passiflora* is a genus of allogamous, mostly self-incompatible plants. However, fruit formation was observed in triploid plants of *P. foetida* and *P*. *cincinnata* (personal communication). Given the limited data on the reproductive aspects of these genotypes, we describe changes that occur during the growth and development of seedlings derived from the crossbreeding of triploid plants, which will help understand the advantages of polyploidization and drive genetic improvement of passion fruit.

## Materials and methods

### Plant material

*P*. *cincinnata* seeds (diploid cytotype) were germinated in black polyethylene plastic bags measuring 28 × 14 cm, filled with Plantmax^®^ substrate, and kept in a greenhouse with controlled irrigation. Triploid plants of *P*. *cincinnata* were obtained following the protocol outlined by Machado et al. (2022), regenerated via in vitro endosperm culture. Only three plants per diploid and triploid cytotype were used.

Triploid plants approximately 10 cm in size were uprooted from the flasks and washed under running water to remove excess culture medium. Subsequently, they were placed in plastic cups (15 × 9 cm) containing moistened Plantmax^®^ substrate with full-strength MS (Murashige and Skoog 1962) solution. The plants were covered with a transparent plastic bag (10 × 20 cm) and kept under a 16-h photoperiod at 26 ± 2 °C for approximately 20 days, followed by their transfer to a greenhouse.

Both diploid and triploid *P*. *cincinnata* plants, whose height reached approximately 25 cm, were transplanted into 30-dm^3^ plastic pots filled with a 3:1 soil and substrate mixture. Cultivation was conducted in a greenhouse with a spacing of 4 m between plants. The training system consisted of vertical trellising with a smooth wire at a height of 1.8 m, without removing the terminal buds and lateral branches. Fertilization was carried out through foliar application at 3–9 L/Ha of a formulation with guaranteed nutrient levels (N = 10%; P_2_O = 10%; K_2_O = 10%; Fe = 0.1%; Zn = 0.1%; Cu = 0.1%; S = 0.3%; and Mn = 0.5% w/w%) once a month during the vegetative phase and every 15 days during the reproductive phase.

**Fig. 1:**
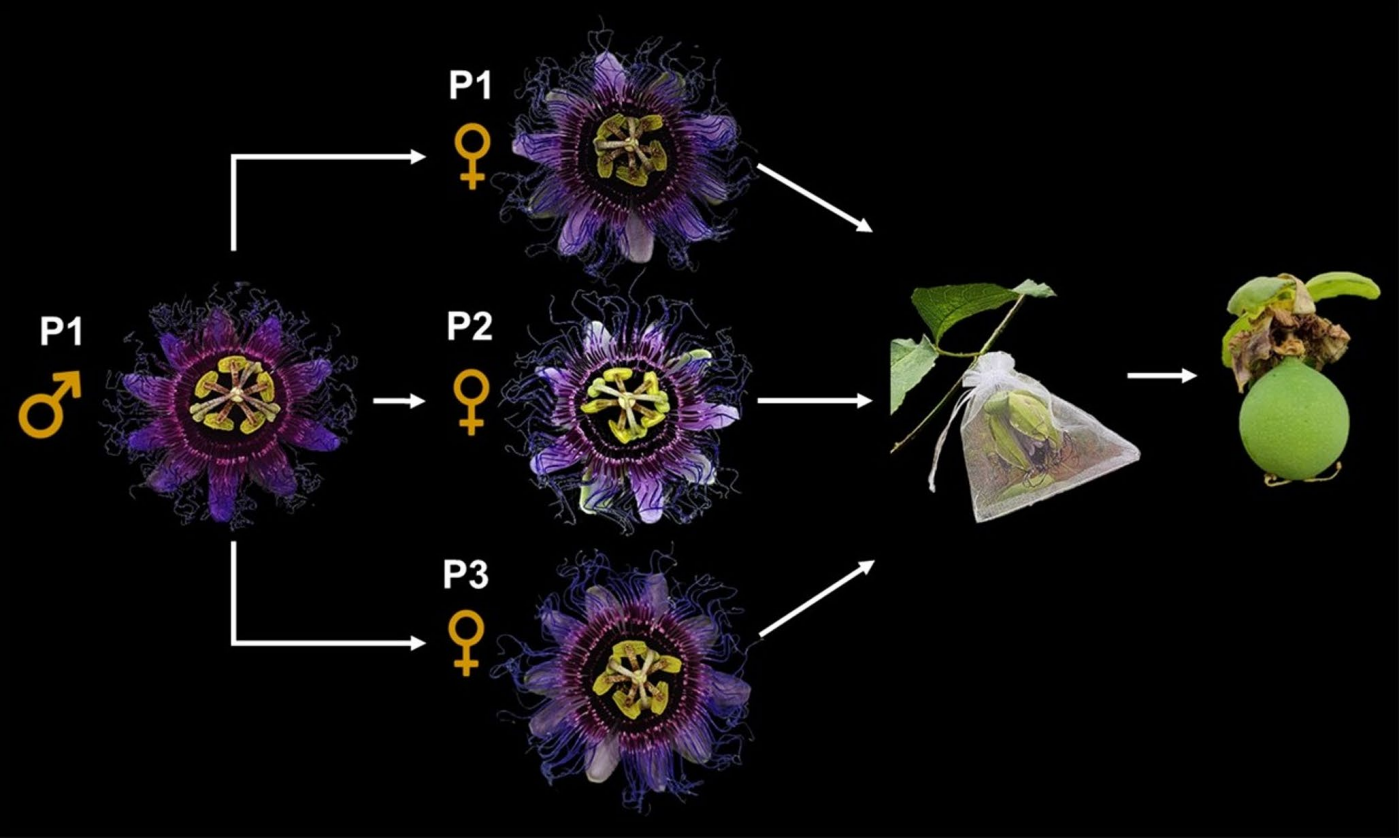
Schematic representation of crossbreeding among three in vitro regenerated triploid plants (P) of *P*. *cincinnata*

For the control group, three diploid plants germinated through seed propagation, and three triploid plants regenerated in vitro through somaticembryogenic pathway were selected. Triploidy was confirmed by estimating the nuclear DNA content via flow cytometry (Doležel and Bartos 2005) and cytogenetics, according to Carvalho and Saraiva (1993, 1997). Crossbreeding among triploids was conducted with three plants: the first cross involved self-pollination triploid 1 × triploid 1 (T1♀ × T1♂), while the subsequent two involved cross-pollination triploid 1 × triploid 2 (T1♂ × T2♀) and triploid 1 × triploid 3 (T1♂ × T3♀). Triploid 1 (T1) served as the pollen-donating male (Fig. 1).

Pollen was transferred to the stigma with forceps by gently rubbing the anther onto the stigma of each flower, after which the flowers were isolated in tulle fabric bags. Fifty flowers per plant were pollinated, for a total of 150 flowers. The same pollination process was used for diploid plants. After 120 days, the fruits produced by the plants were counted, and the seeds were manually extracted, washed under running water, and rubbed on a sieve to remove the aril and mucilage. They were then placed on paper in the shade at room temperature for drying.

### Morphometric evaluation of fruits and seeds obtained from crossbreeding of triploid plants

For morphometric characterization of the fruits, 6 fruits per replicate were randomly selected, for a total of 18 fruits per cytotype (diploid and triploid). Length and width were measured with a digital caliper (Mtx^®^). For seed morphometry, 75 seeds were used for each cytotype, whereas 100 seeds per plant were used for weight quantification. To determine the morphological characteristics of seeds without endosperm, zygotic embryos, malformed seeds, and complete seeds, 300 seeds were used per diploid and triploid cytotypes.

Morphometric characterization of seeds from diploid plants (controls) and those produced from crosses between triploids was conducted using 25 seeds per cytotype for a total of 75 randomly selected seeds. The length, width, and thickness of the seeds were measured with a digital caliper (Mtx^®^). To measure weight, 100 seeds of each cytotype were used and weighed on a precision scale (Shimadzu^®^), after which they were opened using a mini-vise and assessed internally. The average number of seeds lacking endosperm and zygotic embryos, the average number of complete seeds with endosperm and zygotic embryos, the average number of malformed seeds with atrophied endosperm and zygotic embryos, the presence of dehydrated of endosperm and zygotic embryos, and the absence of endosperm and/or embryo were evaluated.

### In vitro germination

In vitro germination of normal seeds, resulting from crossbreeding between triploid and diploid plants, consisted of six replicates of 10 seeds per flask, totaling 60 seeds per cytotype. The seed coats were removed using a mini-vise. Under aseptic conditions in a laminar flow chamber, the seeds were disinfested and immersed in 70% (v/v) ethanol for 3 min, then in 2.5% (v/v) sodium hypochlorite with three drops of Tween-20 for 30 min, and finally in four consecutive rinses in distilled, autoclaved water.

The seeds were cultivated in flasks containing 60 mL medium composed of MS basic salts (Calsson Labs, Smithfield, UT, USA), 0.01% (w/v) myo-inositol, 3% (w/v) sucrose, and 0.8% (w/v) agar (Acumedia^®^; Neogen, Lansing, MI, USA). The cultures were kept in a cultivation room for 30 days without irradiance at 25 ± 2 °C. Next, the in vitro cultures were transferred to a growth room with a photoperiod of 16 h and 36 μmol m^−2^ s^−1^ irradiance (Special Daylight, 20 W; Osram, Barueri, Brazil) at 25 ± 2 °C. At ten days of incubation, germination percentage was evaluated, and at 45 days, shoot length and number of leaves were assessed. Seeds showing radicle protrusions were considered to have germinated.

### Germination speed index

The germination speed index (IVG) was calculated using the Maguire Eq. (1962): IVG = G1/D1 + G2/D2 + … Gn/Dn; where Gn = number of emerged radicles observed at each counting interval and D1, D2, …, Dn = number of days from sowing to the 1st, 2nd, …, and last counting. The germination speed index was evaluated after the seventh day following in vitro inoculation of *P*. *cincinnata* RAS cytotype seeds (adapted from Brazil, 2009).

### Acclimatization of seedlings

Complete plants, measuring approximately 10 cm in height, were washed under running water to remove excess culture medium. Subsequently, they were placed in plastic cups (15 × 9 cm) containing Plantmax^®^ substrate moistened with a full-strength MS-based solution and wrapped in a transparent plastic bag (10 × 20 cm). The cups were covered with transparent plastic bags, and kept under a 16-h photoperiod at 25 ± 2 °C for approximately 20 days. After 5 days, small holes were made in the plastic bag and the cups were kept in the cultivation room for another 5 days before being transferred to a greenhouse.

### Determination of DNA content

Nuclear DNA content was estimated in six seedlings resulting from the cross-pollination of diploid plants, six endosperm-derived plantlets, and ten seedlings resulting from the crossbreeding of triploid plants. Nuclear suspensions were obtained from sections of young leaves using a disposable steel blade in WPB isolation buffer (Loureiro et al. 2007). The suspension was aspirated through two layers of gauze using a plastic pipette and filtered through a Nylon membrane with a 50 μm mesh to remove cell fragments and tissue debris. Subsequently, 25 μL propidium iodide (1 mg mL^−1^) and 5 μL RNase (1 mg mL^−1^) were added to stain the DNA.

After 30 min in a light-protected environment, the samples were analyzed using a CytoFLEX flow cytometer (Beckman Coulter, CA, USA). Three repetitions, amounting to at least 10,000 nuclei, were analyzed. *Pisum sativum* with DNA content of 2C = 9.09 pg (Doležel et al. 1998) was used as an internal standard. Histograms were generated and analyzed using CytExpert 2.0.1. The DNA content (pg) was calculated according to Doležel and Bartos (2005).

2C DNA = Average G1 peak of *P. cincinnata* × 9.09 / Average G1 peak of *P. sativum.*

### Chromosomal analysis

Chromosomal analysis was performed using three seedlings resulting from the cross-pollination of diploid plants, three endosperm-derived plantlets, and three seedlings resulting from the crossbreeding of triploid plants. Approximately 1.5 cm of root apices were collected, treated with 0.003 M 8-hydroxyquinoline (Sigma-Aldrich, St. Louis, MO, USA) for 8 h at 4 °C, fixed in Carnoy's solution (3:1 ethanol: glacial acetic acid), and stored in a refrigerator for 24 h. The cell wall was removed using 20% pectinase (Sigma-Aldrich) and 2% cellulase (Onozuka R-10; Serva, Heidelberg, Germany) for 4 h at 37 °C. The slides were prepared according to Carvalho and Saraiva (1993, 1997) and mounted in Vectashield with DAPI (Vector Laboratories, USA). Metaphase was captured under a BX51 fluorescence microscope equipped with a DP72 camera (Olympus, Japan).

### Stomatal analysis

Diploids produced by seed propagation, triploids resulting from endosperm regeneration, and leaves from the progeny of triploid plants were analyzed. Leaf imprints were made on the middle third of the abaxial side, near the central vein of the leaf. An ethyl-based instant adhesive (Three-Bond Brasil, Diadema, SP, Brazil) was used to fix the epidermal impressions on the slides. Stomatal density (mm^2^), length, and width, as well as pavement epidermal cells density (mm^2^), area, width, and length, were assessed. Three leaves from three plants were used to evaluate density, with five observations per ploidy level (diploid, triploid, aneuploid), totaling 15 observations. The observations were conducted using a light microscope (Bioval^®^) connected to a U-photo camera system with a 10 × eyepiece and a computer running TSView 73.1.7 software.

### Statistical analysis

For the statistical analysis of the collected data, the GraphPad Prism 10 program (GraphPad Software Inc., San Diego, CA, USA) was used, through which the data were subjected to the Shapiro–Wilk test to test normality, for the description of the sample. The data are presented as the means ± standard error. To compare the values, the Tukey test was used; the results were considered significant when *p* < 0.05.

## Results

**Fig. 2:**
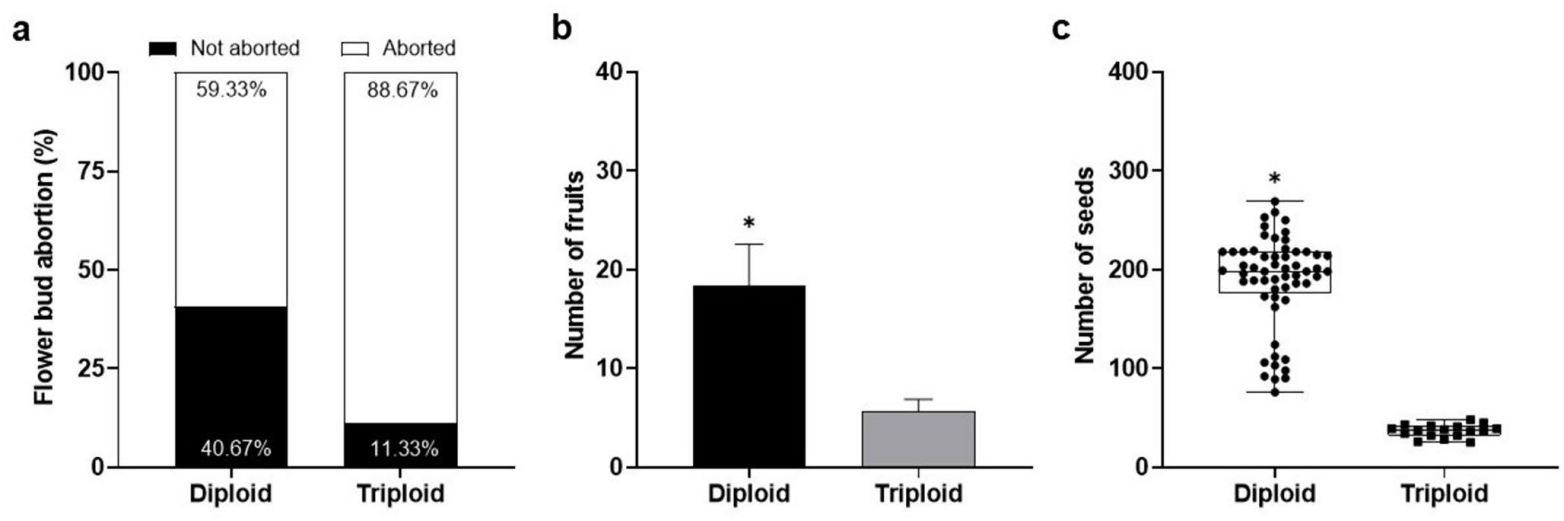
Reproductive performance of diploid and triploid *P. cincinnata* plants. **a** Percentage of aborted and non-aborted flower buds after pollination. **b** Number of fruits produced per cytotype. **c** Number of seeds per fruit in each cytotype. Error bars denote the standard error. Asterisks denote statistical differences between cytotypes for each parameter according to the Tukey test (*p* < 0.05)

Diploid and triploid plants of *P*. *cincinnata* produced floral buds and flowers from September to October, with fruits following in December. The pre-anthesis stage of flowers begins with the gradual separation of sepals, petals, and fringes of the corona, and is accompanied by the exposure of floral organs. Flower anthesis occurred between 5:00 and 6:30 a.m., and closure occurred at approximately 6:00 p.m. After 7 days of pollination, the ovary developed rapidly. The frequency of floral bud abortion was 59.33% in diploid plants and 88.66% in triploid plants (Fig. 2a).

Fruit ripening occurred approximately 120 days after cross-pollination in both diploid and triploid genotypes. Diploid plants produced 18 fruits per plant, with an average of 287 ± 12.30 seeds per fruit; whereas triploid plants produced 6 fruits per plant and, on average, 37 ± 2.21 seeds per fruit (Fig. 2b, c).

### Fruit and seed morphology

**Fig. 3:**
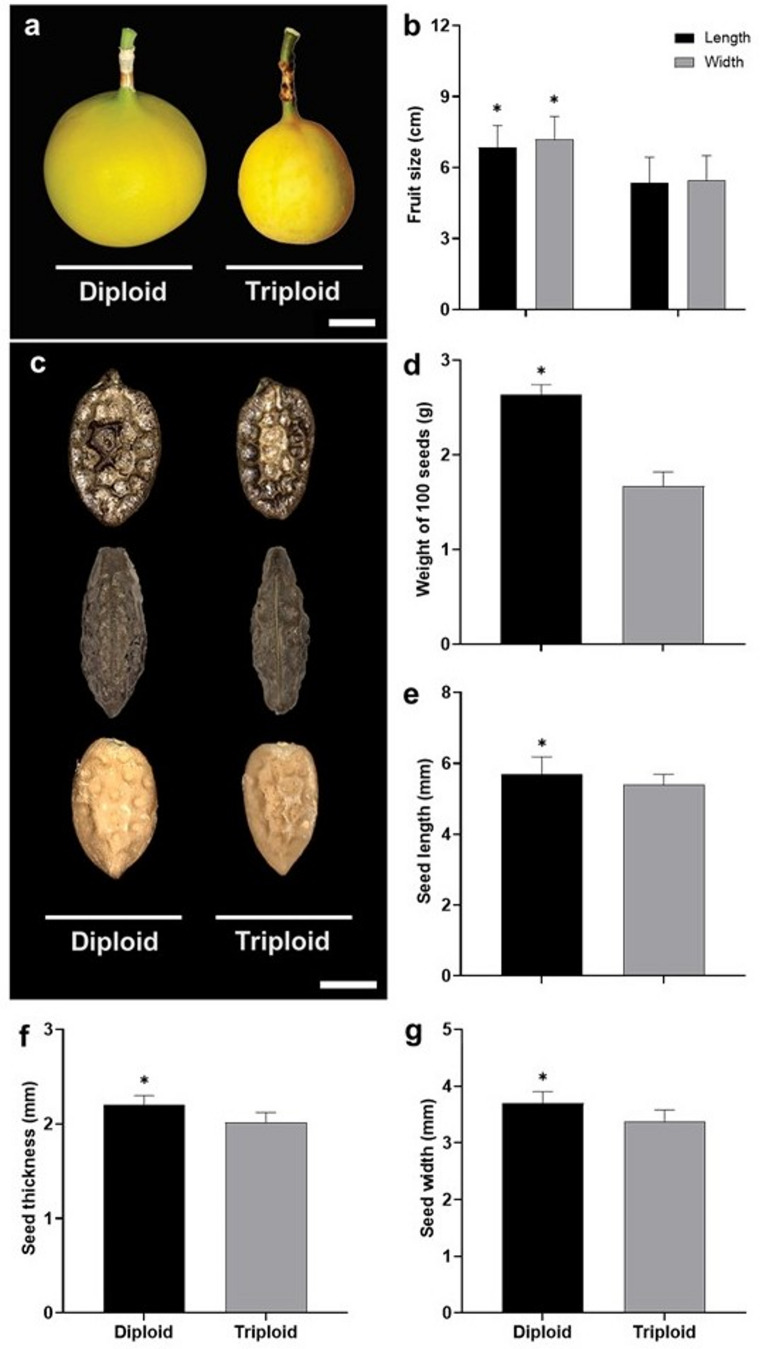
Morphometry of fruit and seeds produced by diploid and triploid *P*. *cincinnata* plants. **a** Front view of fruits. Scale bar: 2 cm. **b** Average length and width of fruits. **c** Front and side views of seeds and front view of endosperms of diploid and triploid cytotypes. Scale bar: 3.8 μm. **d** Average weight of 100 seeds. **e–g** Average length (**e**), width (**f**), and thickness (**g**) of the seeds. Error bars denote the standard error. Asterisks denote statistical differences between cytotypes for each parameter according to the Tukey test (*p* < 0.05)

Diploid plants have more uniform fruits, while triploid plants showed greater variation in size. Fruits from diploid plants averaged 6.84 ± 0.83 cm in length and 7.18 ± 0.87 cm in width; in triploid plants, the fruits averaged 5.34 ± 0.97 cm in length and 5.46 ± 0.92 cm in width (Fig. 3a, b). In general, the seeds of diploid plants exhibited a reticulate surface with deeper grooves, a more rounded shape, and larger dimensions in comparison to the seeds obtained from triploid plants, which showed a reticulate surface with shallower grooves, a more elongated form, and smaller dimensions (Fig. 3c).

Based on a sample of 100 seeds, the average seed weight was 2.64 ± 0.10 g in diploid plants and 1.67 ± 0.15 g in triploid plants (Fig. 3d). The average length and width of the seeds were 5.68 ± 0.50 mm and 3.7 ± 0.20 mm, respectively, in diploid plants, and 5.39 ± 0.30 mm and 3.38 ± 0.20 mm in triploid plants (Fig. 3e, f). Seed thickness was 2.2 ± 0.10 mm in diploids and 2.02 ± 0.10 mm in triploids (Fig. 3g).

**Fig. 4:**
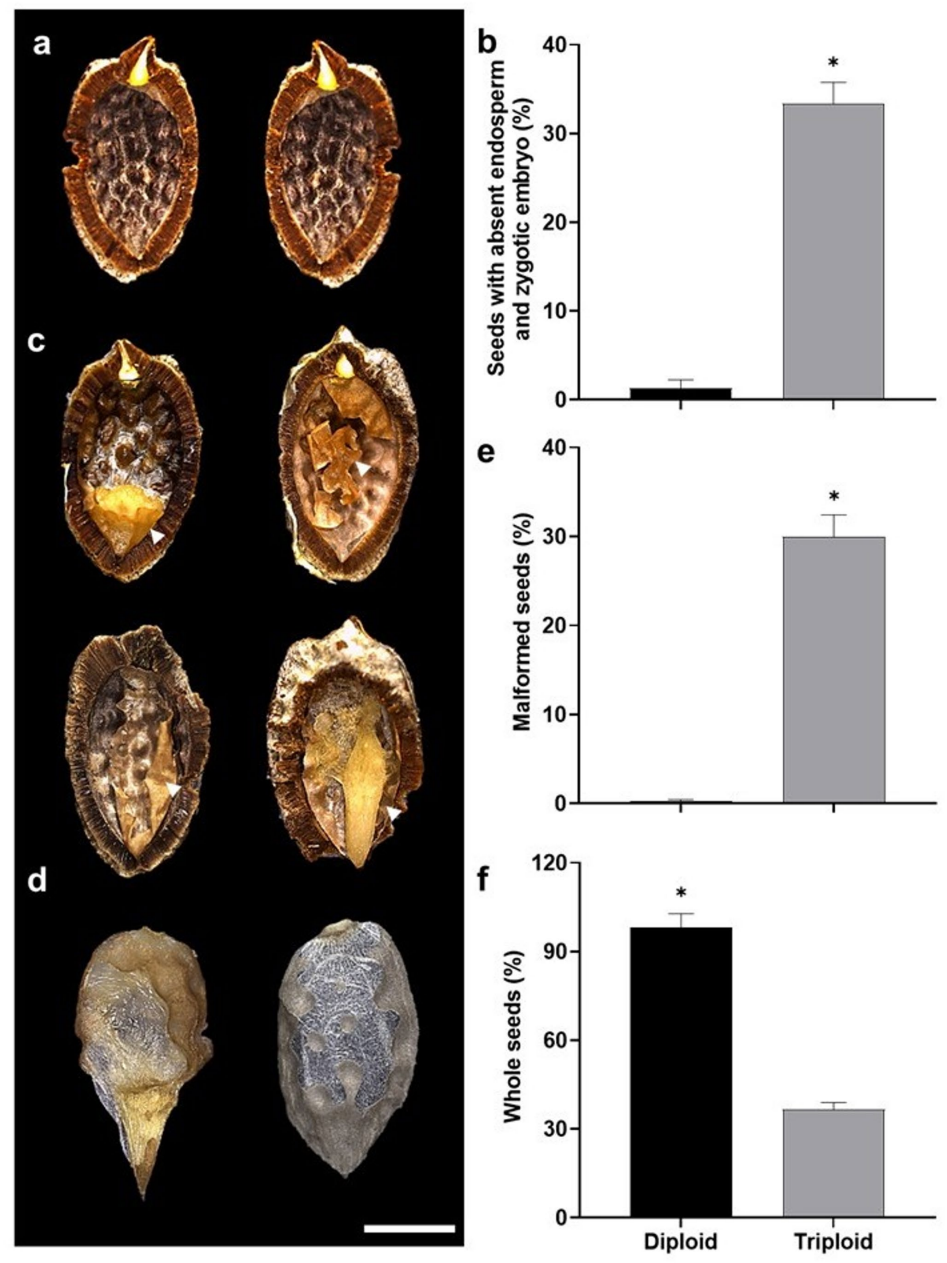
Morphological characteristics of seeds produced by triploid plants of *P. cincinnata*. **a**, **b** Empty seeds. Morphology (**a**) and percentage (**b**) of seeds lacking endosperm and zygotic embryos. **c**–**e** Malformed seeds. Morphology (**c**, **d**) and percentage (**e**) of seeds with malformations, such as atrophy and dehydration of the endosperm (see arrowheads) or deterioration of endosperm and zygotic embryos. **f** Percentage of whole seeds. Bar: 4.6 μm. Error bars denote the standard error. Asterisks denote statistical differences between cytotypes for each parameter according to the Tukey test (*p* < 0.05)

Evaluation of 300 seeds per cytotype revealed that 1.33% ± 0.92% of diploid seeds and 33.33% ± 2.45% of triploid seeds lacked the endosperm and zygotic embryos (Fig. 4a, b). Seed malformations were detected in 0.33% ± 0.10% of diploid seeds and 30% ± 2.34% triploid seeds (Fig. 4c–e). Common malformations included atrophy, dehydration, and deterioration of the endosperm, as well as deterioration of the zygotic embryo (Fig. 4c, d). Whole seeds accounted for 98.33% ± 4.50% in the diploid cytotype and 36.66% ± 2.30% in the cytotype triploid (Fig. 4f). Overall, these structural and developmental alterations were mainly observed in triploid cytotypes, indicating a higher frequency of defective seed formation associated with increased ploidy level.

### Seed germination and seedling development

**Fig. 5:**
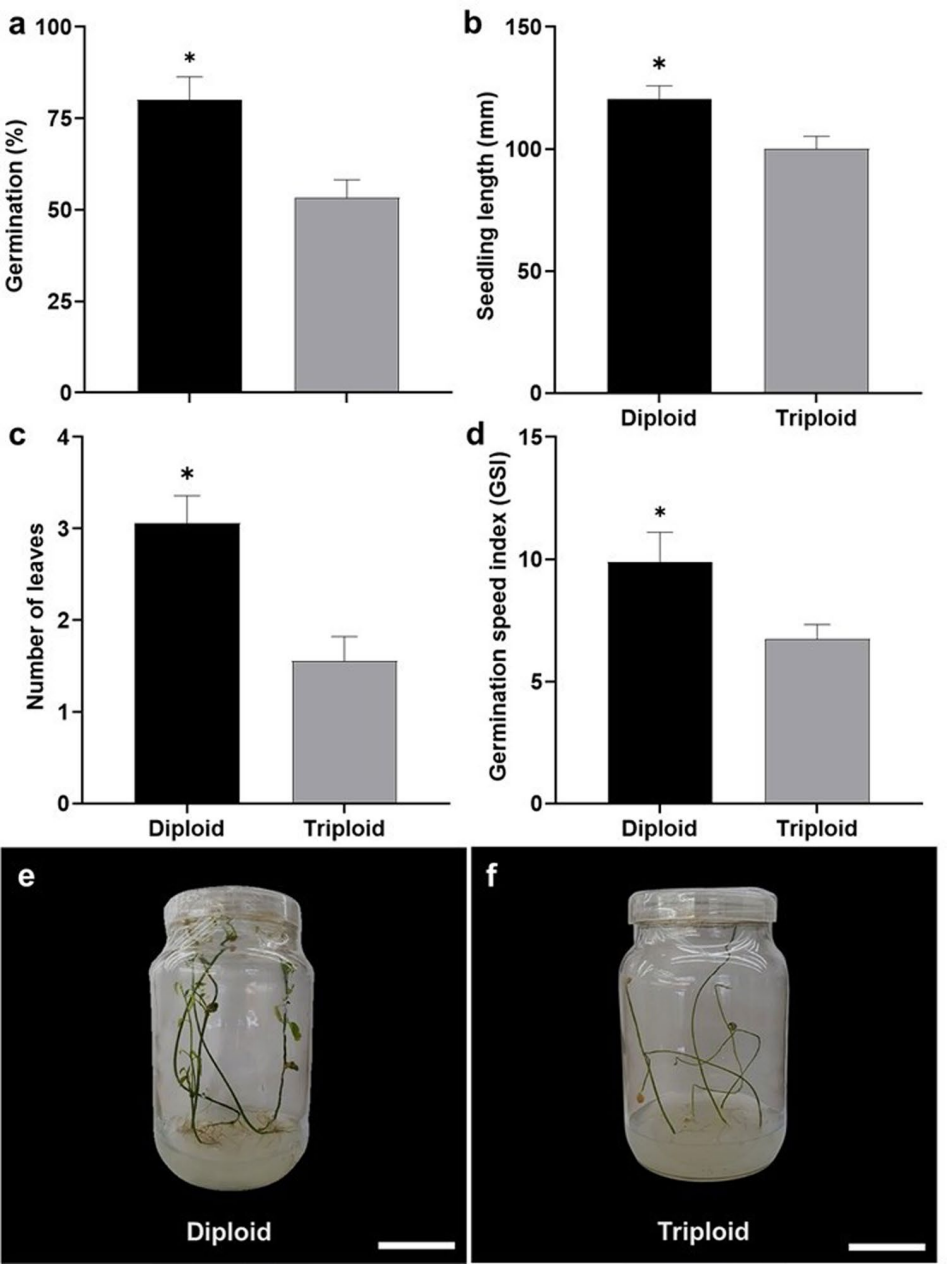
In vitro germination of diploid and triploid cytotypes of *P. cincinnata*. **a** Percentage of in vitro germination. **b** Average length of diploid and triploid seedlings. **c** Average number of leaves. **d** Germination speed index. **e** Diploid seedlings of *P*. *cincinnata*. **f** Seedlings resulting from the cross between triploid plants. Bar: 130 mm. Error bars denote the standard error. Asterisks denote statistical differences between cytotypes for each parameter according to the Tukey test (*p* < 0.05)

Seeds from diploid plants cultured in vitro achieved a germination rate of 80% ± 6.30%, whereas seeds from crosses between triploid plants had a germination rate of 53.3% ± 4.90% (Fig. 5a). At 45 days after sowing, average seedling length was 120.34 ± 5.53 mm in diploids and 100.02 ± 5.12 mm in triploids (Fig. 5b). The highest average number of leaves was observed in diploid seedlings (3.06 ± 0.30 leaves), whereas the triploid progeny presented an average number of leaves of1.56 ± 0.26 (Fig. 5c). Diploid seeds showed a higher germination speed index (9.9 ± 1.20) compared to the progeny of triploid plants (6.72 ± 0.60) (Fig. 5d). In addition, seeds from the cross of triploid plants germinated less uniformly compared to those from diploid plants, (Fig. 5e, f).

### Seedling DNA content

**Fig. 6:**
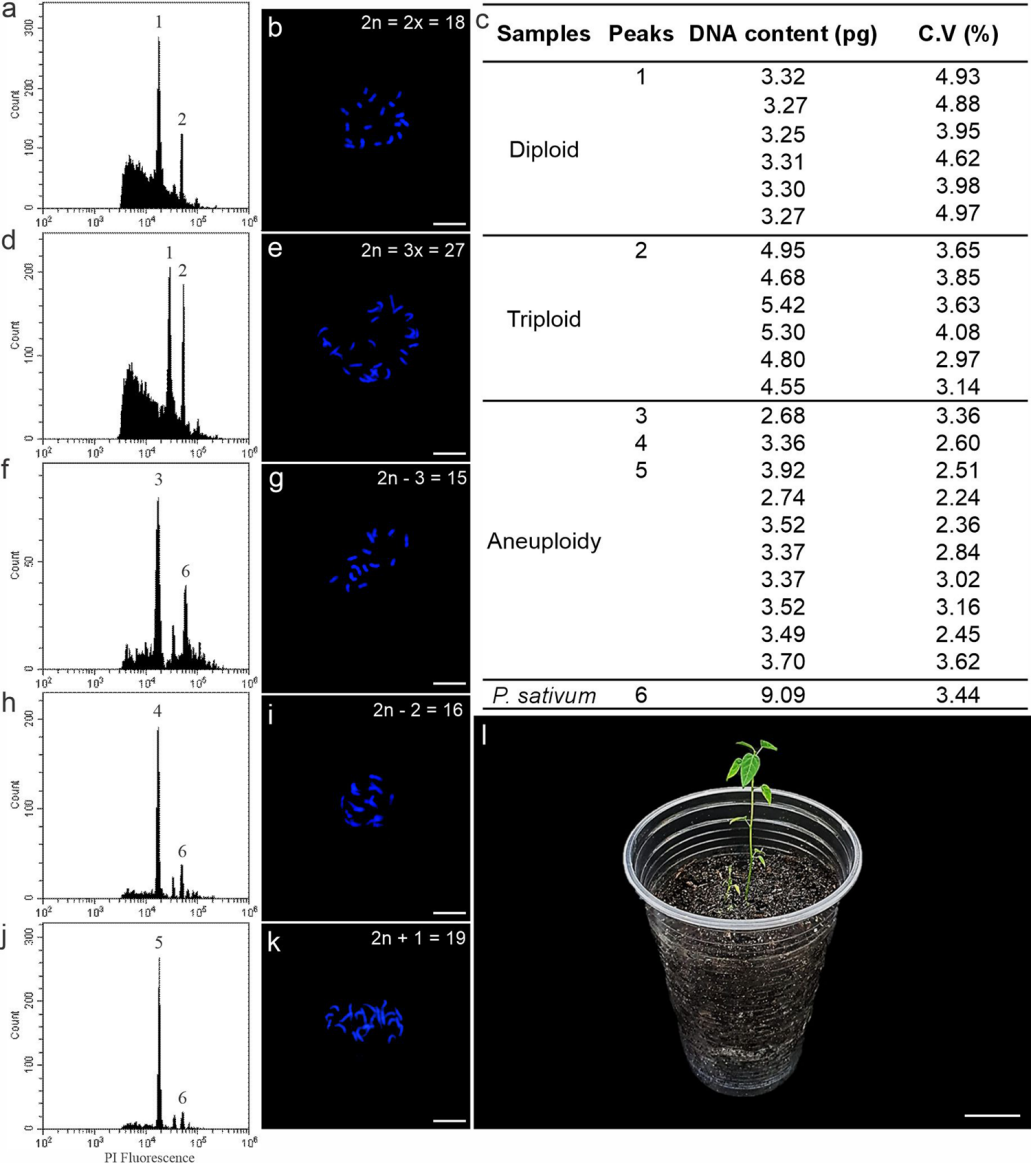
Nuclear DNA and chromosome content of diploid, triploid, and aneuploid cytotypes of *P. cincinnata*. **a** Histogram comparing nuclear DNA of diploid seedlings and *P*. *sativum* (standard). **b** Chromosomal number of diploid plants 2n = 18. **c** Nuclear DNA content (pg) and coefficients of variation (CV%) for diploid, triploid, and aneuploid samples. **d** Histogram comparing nuclear DNA of diploid seedlings and triploid endosperm-regenerated plants. **e** Chromosomal number of triploid plants 2n = 27. **f**, **g** Histogram (**f**) comparing nuclear DNA of aneuploid plant and *P. sativum*, with chromosomal number 2n = 15 (2n-3) (**g**). **h**, **i** Histogram (**h**) comparing nuclear DNA of aneuploid plant and *P*. *sativum*, with chromosomal number 2n = 16 (2n-2) (**i**). **j**, **k** Histogram (**j**) comparing nuclear DNA of aneuploid plant and *P*. *sativum*, with chromosomal number 2n = 19 (2n + 1) (**k**). Bar: 5 µm. **l** Aneuploid seedling acclimatized in the greenhouse. Scale bars: 10 µm (**b**, **d**, **f**, **h**, **j**); 125 mm (**l**)

Diploid plants contained an estimated 3.29 ± 0.19 pg of nuclear DNA, with a coefficient of variation of 4.56% and 2n = 2x = 18 chromosomes (Fig. 6a–c). In contrast, for triploid plants, the corresponding value was 4.95 ± 0.10 pg, with a coefficient of variation of 3.55% and 2n = 3x = 27 chromosomes (Fig. 6c–e). The ten seedlings obtained from the crossbreeding between triploids had 3.37 ± 0.23 pg of nuclear DNA, with a coefficient of variation of 2.84%, and were considered putative aneuploids (Fig. 6c, f, h, j). Crossbreeding triploid plants produced seedlings with variable chromosome numbers. Among the five seedlings evaluated through cytogenetic analysis, one exhibited 2n = 15 (2n – 3) chromosomes (Fig. 6g), another showed 2n = 16 (2n – 2) (Fig. 6i), and three seedlings presented 2n = 19 (2n + 1) chromosomes (Fig. 6k). The aneuploid seedlings were acclimatized in the greenhouse (Fig. 6l).

### Stomata analyses

**Fig. 7:**
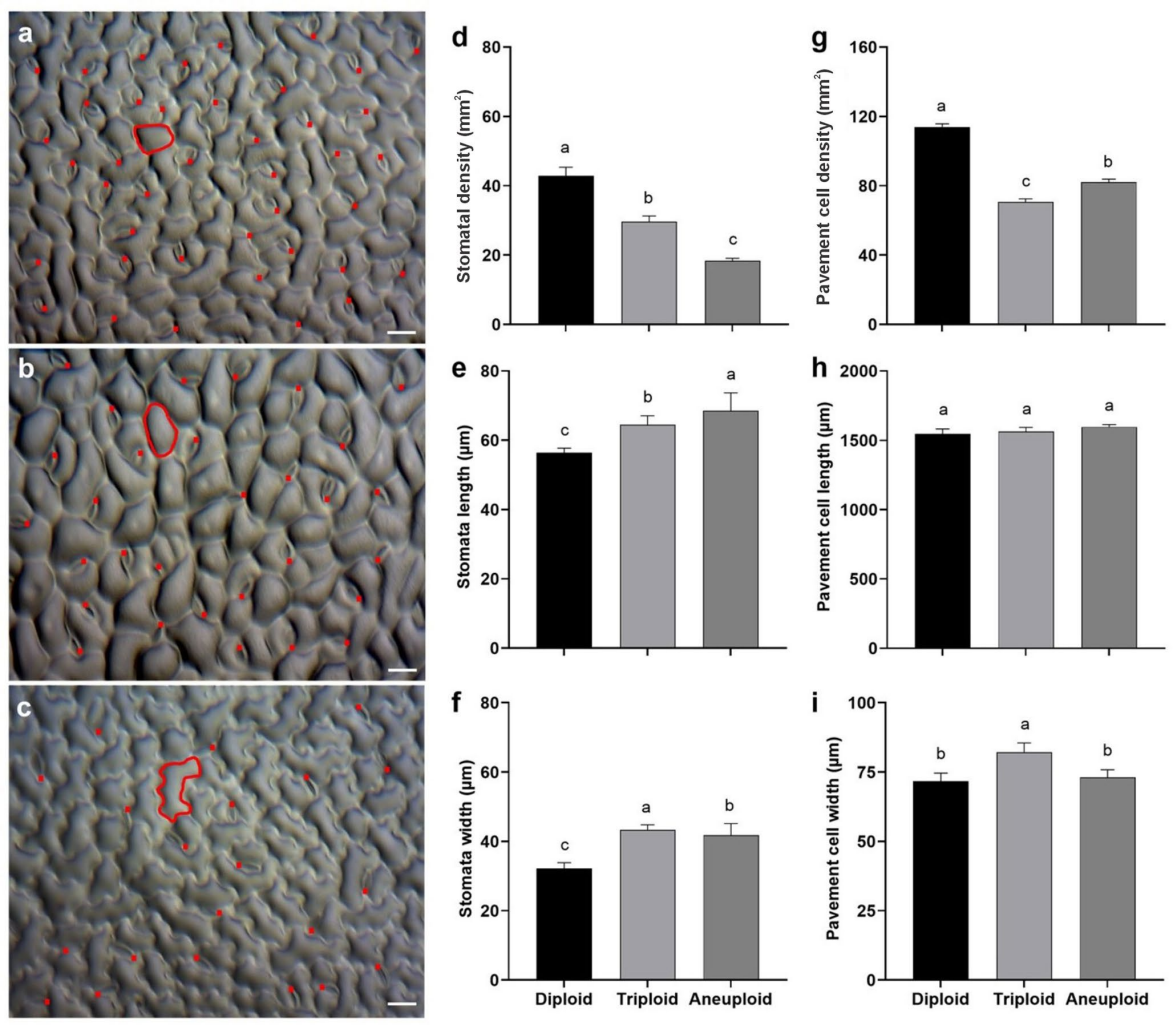
Characterization of the epidermis in diploid, triploid, and aneuploid *P. cincinnata* plants grown in the greenhouse. **a–c** Leaf epidermis of a diploid (**a**), triploid (**b**), and aneuploid **c** seedlings. Bar: 100 μm. **d** Stomatal density (mm^2^). **e, f** Stomata length (**e**) and width (**f**). **g** Pavement epidermal cells density (mm^2^). **h**, **i** Length (**h**) and width (**i**) of pavement epidermal cells. Error bars denote the standard error. Means followed by different letters for each parameter refer to statistical difference according to the Tukey test (*p* < 0.05)

Stomatal density, length, and width differed significantly among diploid, triploid, and aneuploid cytotypes (Fig. 7a–c). The highest stomatal density was observed in diploid plants (42.9 ± 2.42 stomata per mm^2^), followed by triploid plants (29.6 ± 1.69 stomata per mm^2^) and aneuploid plants (18.4 ± 0.66 stomata per mm^2^) (Fig. 7d). The average stomata length was 56.43 ± 1.24 µm in diploids, 64.44 ± 2.61 µm in triploids, and 68.49 ± 5.17 µm in aneuploid plants (Fig. 7e). In contrast, stomata width was 43.33 ± 1.61 µm in triploids, 41.77 ± 1.43 µm in aneuploids, and 32.22 ± 3.33 µm in diploids (Fig. 7f).

Differences in density and width of pavement epidermal cells across cytotypes were also observed. There were 70.7 ± 1.79 pavement cells per mm^2^ in triploids, followed by 81.1 ± 1.71 cells per mm^2^ in aneuploids and 114 ± 1.77 cells per mm^2^ in diploids (Fig. 7g). The average length of pavement epidermal cells was 1596.09 ± 16.73 µm in aneuploid plants, 1562.07 ± 31.31 µm in triploid plants, and 1545.64 ± 35.14 µm in diploid plants (Fig. 7h). The width of pavement epidermal cells in triploid plants was 82.15 ± 3.38 µm, which was statistically different from that of diploid (71.65 ± 2.93 µm) and aneuploid (73.14 ± 2.68 µm) plants (Fig. 7i).

## Discussion

Meiotic disturbances are common in triploid plants, compromising gamete viability and leading to reduced fertility, seed sterility, and fruit abortion (Grosser et al. 2011; Ramakrishnan et al. 2025; Sattler et al. 2016; Wang et al. 2016). In the present study, fruits and seeds were produced after cross-pollination of triploid *P*. *cincinnata*, although at significantly lower rates than those observed in diploid plants. This reduced fruit and seed set likely results from meiotic irregularities and the consequent formation of aneuploid gametes, which are frequently inviable and have been reported as a typical outcome of polyploid meiosis (Cheng et al. 2005; Jauhar 1970; Mohr 1986; Svačina et al. 2020). The occurrence of unbalanced chromosomes during meiosis, such as trivalents, bivalents, and univalents, contributes to the production of unbalanced or aneuploid gametes, thereby decreasing fertility and viable seed formation (Cuenca 2010; Otto and Whintoon 2000; Wang et al. 2016). Another factor limiting the reproductive capacity of *P. cincinnata* is self-incompatibility, which prevents the formation of the pollen tube by pollen grains from the same flower or individual (Aular et al. 2004). Fertilization thus depends on the presence of different genotypes. Nevertheless, fruit formation has been reported in self-pollination tests, albeit at a much lower rate (7.6%) compared to cross-pollination (63.2%) (Aular et al. 2004; Costa et al. 2008). In *P*. *cincinnata*, the combined effects of self-incompatibility and meiotic instability appear to play a central role in reducing fruit and seed set in triploid plants. Although fruit formation occurred after cross-pollination, the number of viable seeds was substantially lower than in diploids. This reduced fertility likely results from irregular meiotic pairing in triploids, which leads to the production of unbalanced gametes and, consequently, the low fruit and seed yield observed in triploid cytotypes of *P*. *cincinnata*.

Low fertility does not imply a disadvantage in polyploid ornamental species, as fruit development shortens the flowering period and reduces ornamental value (Manzoor et al. 2019; Schifino-Wittman and Dall’agnol 2003). In the genus *Passiflora*, the production of triploid plants for ornamental purposes has attracted increasing attention. Ploidy levels can lead to phenological and morphological consequences that may be of commercial interest, as observed in *P*. *cincinnata* (Machado et al. 2022) and *P*. *foetida* (Mikovski et al. 2021). The generation of larger vegetative or reproductive organs has increased interest in passion fruit as an ornamental plant.

The seeds produced by triploids of *P*. *cincinnata* exhibit smaller morphometric parameters but a high incidence of missing or improperly formed endosperm and zygotic embryos. These differences in the size and weight of triploid seeds are related to reduced development or premature completion of the endosperm (Esen and Soost 1975). According to Kaiser et al. (2016), variation in seed size is an indicator of adaptability to environmental changes in ripening, genetic factors, pollination rate, nutrient availability, water, and light.

The endosperm is a storage tissue in angiosperms, whose role is to supply nutrients to the growing embryo until the seedling becomes autotrophic. Successful germination depends on the proper formation and development of the endosperm (An et al. 2020). Here, triploid seeds of *P*. *cincinnata* exhibited endosperm and zygotic embryo degradation, becoming completely inviable, whereas others matured but showed signs of deterioration during storage.

Loss of seed vigor hampers the ability to produce normal seedlings. This loss is caused by physical, physiological, and biochemical changes during seed development (Krzyzanowski and Neto 2001; Peske et al. 2003). The low vigor of triploid seeds affected physiological traits, including germination rate, seedling growth, and development. The germination rate of triploid seeds was only 53.3%, significantly less than the 80% of diploid seeds. Low seed vigor may be responsible for uneven in vitro germination and slow seedling growth and development.

Crossbreeding between triploids resulted in the production of aneuploid plants of *P*. *cincinnata* with a nuclear content of 3.37 pg and variable chromosomal numbers, including 2n – 3 = 15, 2n – 2 = 16, and 2n + 1 = 19. Gain or loss of chromosomes affects morphophysiological characteristics (Birchler 2013; Sheltzer and Amon 2011; Singh 2020). This imbalance in gene dosage leads to phenotypic alterations such as delayed vegetative development, low vigor, non-uniform germination, and death in the juvenile phase, as observed in the progeny of triploid *P*. *cincinnata*. Hybrids from the cross between *Passiflora sublanceolata* (2n = 22) and *P*. *foetida* (2n = 22) exhibited aneuploidy (2n = 22 − 2), probably because of centromere function suppression and asynchrony of the cell cycle (Santos et al. 2012).

Gene expression in aneuploid plants is affected by gene dosage imbalances (Zeng et al. 2020; Zhu et al. 2015). Changes in expression patterns in aneuploids correlate with minor alterations in genes located in duplicated chromosomal regions, and characterized by a 1.5-fold increase in gene dosage (Makarevitch and Harris 2010; Zhu et al. 2015). Given a complex network of interactions, such alterations result in significant changes in the expression of genes related to morphological, anatomical, cytological, and physiological traits (Birchler 2013; Singh 2020). The identification of gene regions associated with morphological and quantitative traits in aneuploid individuals will enable the identification of genes responsible for agronomic traits important for plant breeding (Birchler 2013; Singh 2020).

Stomatal analyses of *P*. *cincinnata* revealed that aneuploid plants had fewer stomata compared to triploid plants and less than half of those in diploid plants. Moreover, their stomata were larger compared to those of diploid and triploid plants. Instead, ordinary epidermal cells presented a lower density in triploid plants, followed by aneuploids and diploids. Similar variations in stomatal density in *Passiflora* polyploids were found also in *P*. *edulis* (Antoniazzi et al. 2018), *P*. *cincinnata* (Silva et al. 2020) and *P*. *foetida* (Mikovski et al. 2021).

In summary, morphological, physiological, and biochemical alterations were observed in triploid *P. cincinnata* plants. While these plants were able to produce fruits and seeds, their progeny exhibited clear signs of aneuploidy, with a nuclear DNA content of 3.37 pg and variable chromosome numbers (2n − 3 = 15, 2n − 2 = 16, and 2n + 1 = 19). The genetic material of aneuploids is a resource for gene mapping, which will facilitate the modification or production of new genotypes, with economically relevant agronomic characteristics.

## Data Availability

The data supporting the findings of this study are availablefrom the corresponding author upon reasonable request.
